# Spontaneous Coronary Artery Dissection (SCAD) from an Interventionalist Perspective

**DOI:** 10.1007/s11886-023-02019-w

**Published:** 2024-01-18

**Authors:** Nichole Brunton, Patricia J. M. Best, Kimberly A. Skelding, Emily E. Cendrowski

**Affiliations:** 1 Department of Cardiovascular Diseases, Mayo Clinic College of Medicine, 200 1st St SW, Rochester, MN 55905 USA; 2 Department of Cardiovascular Disease, Confluence Health, Wenatchee, WA USA

**Keywords:** Spontaneous coronary artery dissection, Extracoronary vascular abnormalities, Percutaneous coronary intervention, Women’s cardiovascular health

## Abstract

**Purpose of Review:**

Spontaneous coronary artery dissection (SCAD) is an increasingly recognized cause of acute coronary syndrome (ACS), particularly among women < 50 years of age. Here, we aim to review the pathogenesis of SCAD, discuss SCAD as an initial manifestation of systemic arterial disease, and highlight invasive strategies as well as unique challenges in the care of women with SCAD.

**Recent Findings:**

A paradigm shift has occurred in the care of SCAD patients in the past decade as recommendations for conservative management have become widespread. Invasive interventions are reserved for patients with hemodynamic compromise or active ischemia due to increased periprocedural complications and failure rates. Certain patient populations have been identified for larger territory infarcts and proximal disease including patients with known connective tissue disease, premenopausal women, and patients with pregnancy-associated SCAD (P-SCAD).

**Summary:**

Current recommended management of SCAD is conservative. Despite a growing awareness of SCAD and its known association with systemic arteriopathies in women, evidence-based data remains scarce. Future studies focused on identifying genetic factors, optimal medical therapy after SCAD, and techniques to minimize interventional complications are needed.

## Introduction

Spontaneous coronary artery dissection (SCAD) is an increasingly recognized etiology for acute coronary syndrome (ACS) in the young, accounting for 1–4% of all ACS cases [1, 2]. While SCAD has been reported in both sexes, the vast majority of cases occur in middle-aged women from ages 44 to 53 years old in the absence of traditional cardiovascular risk factors [1, 3–5]. While prior population studies report SCAD as a rare etiology for myocardial infarction, recent studies demonstrate SCAD as the cause of 35% of all ACS events in women under the age of 50 years old [5, 6••, 7].

Within the past 5 years, multiple expert consensus statements have been published promoting conservative management for stable SCAD patients, including guidelines from the American College of Cardiology (ACC) and American Heart Association (AHA) [5, 6••]. These recommendations emphasize the need to treat SCAD as a heralding signal to systemic arterial disease. Despite this, screening for extracoronary vascular abnormalities has not been fully adopted. Additional studies are needed to determine the genetic predisposition for SCAD and understand the molecular mechanisms responsible for this pathology.

To date, most data on SCAD is retrospective, with only a few cohort studies available to support currently accepted conservative management. Little data exists to provide insight into diagnostic strategies or optimal management for patients with unstable, recurrent, or multivessel SCAD.

## SCAD Pathophysiology

SCAD is the nonatherosclerotic, nontraumatic formation of an intramural hematoma (IMH) in the tunica media of an epicardial coronary artery [1, 5]. As the IMH propagates, the intima dissects from the supporting arterial wall, leading to a narrowing or occlusion of the vessel’s true lumen. This process can occur with or without an intimal tear. Ultimately, this leads to acute myocardial ischemia (AMI) due to luminal compression due to the IMH or dissection flap.

Two prevailing theories exist for the pathogenesis of SCAD, being the “outside-in” and “inside-out” hypotheses. Current literature and advances in imaging favor the “outside-in” model; proposing IMH develops sporadically within the tunica media, potentially from abnormalities within the vasa vasorum [1, 5, 8, 9].

SCAD occurs in patients with vulnerable arterial substrate with an accompanying trigger. Described triggers for SCAD include stress (physical or psychological), use of illicit substances, underlying arteriopathies, and hormonal factors such as pregnancy [1, 5, 6••]. Additional infrequent, but important, triggers include inflammatory disorders such as inflammatory bowel disease or systemic vasculitides such as Takayasu arteritis [1, 10]. Multivessel SCAD or SCAD with concurrent other arterial dissections also support the concept of the systemic triggering factors for the event. These triggers have yet to be full understood.

## Hormones and SCAD

Sex hormones have been implicated in the pathogenesis of SCAD due to the strong predilection for women and its association with pregnancy [5]. However, this association is not well understood. Prior reports show similar prevalence of SCAD for both premenopausal and postmenopausal women. Additionally, SCAD prevalence in cohort studies appears similar regardless of pregnancy history [1, 5, 11, 12].

While pregnancy-associated SCAD (P-SCAD) accounts for a minority of all SCAD cases, ranging from < 5 to 17%, it represents a common cause of ACS events in pregnancy [2, 5, 13, 14]. Most P-SCAD events occur within the first week postpartum, at a time of significant hormone change. Some have postulated the rapid decline in hormone levels creating an “estrogen withdrawal” which may be the precipitating trigger [2]. It remains uncertain if absolute hormone values or the fluctuation in estrogen/progesterone levels impact the risk of SCAD [2, 5]. Little data exists to support the safety of future pregnancy after P-SCAD event; avoidance of pregnancy following SCAD has been generally suggested [15].

## SCAD and Systemic Vasculopathy: A Manifestation of Systemic Disease

In patients presenting with SCAD, underlying systemic vasculopathy must be considered. Prior large cohort studies and registry data have solidified the association of SCAD with fibromuscular dysplasia (FMD), with reported rates of FMD occurring typically from 45 to 77% in patients screened for extracoronary vascular abnormalities (EVAs) [5, 15–17]. Furthermore, in patients screened for EVAs, cerebral aneurysm was identified in 7 to 22% of patients [11, 16, 19]. This led to the 2018 AHA Scientific Statement recommending that SCAD patients be screened for EVAs [20–22].

Despite this recommendation, comprehensive head-to-pelvis screening for EVA remains poor, even at tertiary referral centers with a recently reported rate of < 20% [6••]. While there is a lack of satisfactory data to determine the long-term benefits of identifying EVA, this should still be pursued. This is particularly important for women who are pregnant or desiring future pregnancy due to implications for family planning.

Research to identify genetic determinants of SCAD is ongoing. Not infrequently, patients presenting with SCAD may undergo screening for connective tissue diseases and aortopathies such as Loeys-Dietz, Ehlers-Danlos, and Marfan syndrome. While a minority of patients will have an identified pathogenic variant, this carries significant implications for the patient, family members, and plans for future pregnancies [5]. Patients with a known personal or family history of vasculopathy presenting with ACS should raise clinical suspicion for SCAD.

## Clinical Associations for SCAD with Severe Features

Though the pathogenesis of SCAD is still being explored, studies have signaled that certain patient subsets tend to present with more severe clinical features: premenopausal women, those with concurrent vasculopathies, and P-SCAD.

A study utilizing the Spanish Registry on SCAD demonstrated that premenopausal women more often experienced an ST-segment elevation myocardial infarction (STEMI) compared to postmenopausal women. When compared angiographically, premenopausal women more frequently had proximal vessel disease, involvement of multiple coronary segments, and typically with longer lesion lengths [23]. This occurred despite lower rates of traditional cardiovascular risk factors when compared to postmenopausal women in the registry [23]. This likely correlates to additional studies reporting larger magnitude events occurring at younger ages given a higher likelihood of being premenopausal [24].

An additive risk for large myocardial infarct, typically as a consequence of STEMI on initial presentation, is the presence of connective tissue disorder. Patients with hypermobility, defined as a Beighton score of > 4, were found to incur larger chronic infarcts on cardiac magnetic resonance imaging following SCAD [23]. These findings were consistent with the Canadian SCAD cohort study which reported higher 30-day MACE events in SCAD survivors with known connective tissue disorder [11].

Patients with P-SCAD more commonly present with STEMI [25]. This may be attributed to more frequent multivessel or left main coronary artery involvement, along with higher rates of left ventricular dysfunction and cardiogenic shock [5, 25]. In one study by Tweet et al., left main coronary artery involvement occurred in 24% of patients compared to 5% in non-pregnancy associated SCAD (NP-SCAD) [25]. In another observational study, while P-SCAD was not considered an independent predictor of large chronic myocardial infarction (> 10%), patients with P-SCAD were at increased risk for proximal disease compared to NP-SCAD [24].

## Angiographic Diagnosis of SCAD

Since SCAD patients present with symptoms of acute coronary syndrome (ACS), the most utilized diagnostic tool is coronary angiography. The left anterior descending (LAD) artery is the most frequently affected vessel in SCAD, though multivessel involvement can occur in up to 15% of cases [1, 26•]. The Yip-Saw classification outlines three angiographic subtypes of SCAD, distinguishing between these subtypes provides valuable insight to assist with clinical decision-making and management.

Type 1 is characterized by a dual-lumen sign, occurring when the true and false lumen can be simultaneously visualized due to a communicating fenestration [26•]. This finding is pathologically distinct from the other Yip-Saw subtypes as it represents a later stage of dissection and often signifies a lower risk for progression [21, 26•]. Meanwhile, only the true lumen can be appreciated in type 2 and 3 lesions. Type 2 is the most common angiographic finding, described as a tapering, diffuse, and smooth stenosis with a typical lesion length of > 20–30 mm [21, 27]. These lesions will not improve with the administration of intracoronary nitroglycerin [27]. Type 3 lesions, reported in a minority of cases, appear to be focal and can be easily misdiagnosed as atherosclerotic disease [27]. Pretest probability for atherosclerotic disease is key in identifying SCAD in patients presenting with type 3 lesions [1, 26•]. The addition of a type 4 lesion, being complete vessel occlusion, has been proposed to the current classification system, and further modifications may be needed as SCAD becomes increasingly identified on angiogram [26•].

At the time of angiogram, a prior cohort study by Saw et al. demonstrated that culprit lesions had an average stenosis of 78.7% with a measured dissection length of 45–48 mm, representing a longer lesion length when compared to atherosclerotic disease [16, 26•]. Thrombolysis In Myocardial Infarction (TIMI) flow 3 was present in slightly over 50% of the cases on initial angiography [27]. Another angiographic review demonstrated increased coronary artery tortuosity, defined as the presence of three or more consecutive vessel curvatures of 90–180° when compared to patients presenting for ACS from alternative etiologies [18]. Increased vessel tortuosity was most frequently appreciated within the left circumflex artery and was observed more frequently in non-culprit arteries [18].

Angiographic findings must be considered in the context of patient age, gender, and medical history. Additional angiographic clues to assist in the diagnosis of SCAD include otherwise minimal atherosclerotic disease and vessel tortuosity [18]. Differentials that must be considered include iatrogenic vessel dissection, atherosclerotic disease, coronary vasospasm, takotsubo cardiomyopathy, myocardial bridging, and embolization [26•]. When the diagnosis is uncertain, alternative intracoronary imaging modalities such as optical coherence tomography (OCT) or intravascular ultrasound (IVUS) may be helpful [9, 26•, 28, 29••].

## Current Recommendations for Conservative Management

Acute management of SCAD is focused on maintaining myocardial perfusion distal to the lesion site [5]. The use of thrombolytic therapy has been reported to adverse events due to IMH extension and therefore is not recommended [5]. Although no randomized control studies for the management of SCAD exist, recent cohort studies support conservative management when feasible [6••]. Guidelines from the major cardiovascular societies such as AHA, ACC, and EHS have successfully increased awareness of SCAD and led to a widespread practice of conservative management, previously occurring in 35% of cases in 2013 to 89% in 2019 [6••].

Medical therapy for SCAD initially begins with standard therapy for ACS including anticoagulation and antiplatelet therapy. The benefit of this strategy is unclear as there is a theoretical risk for IMH extension and dissection propagation due to further bleeding, though this is balanced by the risk for intraluminal thrombosis related to luminal narrowing [1, 5]. After the diagnosis of SCAD, anticoagulation should be discontinued unless there is a concurrent alternative indication for therapy. Similarly, no data is currently available to support the use of antiplatelet therapy following SCAD. In both instances, there is concern for bleeding potentiation without a clear benefit for patients [5, 20].

The mainstay of medical treatment after SCAD diagnosis includes guideline therapy for patients with reduced ejection fraction when warranted. Beta-blocker therapy may be also warranted if ventricular ectopy occurs. Single-center studies have espoused the routine use of beta-blocker therapy to reduce the risk of recurrence. There has been extrapolation of beta-blocker benefits from patients treated for atherosclerosis and aortic dissection; however, larger studies are necessary to validate this theory [1, 21, 30]. Clinical trials are currently ongoing to examine the use of beta-blockers, antiplatelet therapies, and angiotensin receptor blockers in SCAD management [31].

The rationalization for a conservative approach is threefold. First and foremost, the vast majority of medically managed SCAD lesions demonstrate resolution on subsequent angiographic imaging, typically achieving resolution by 1 month following the index event [1, 27, 32]. Secondly, recurrent SCAD typically impacts a separate coronary segment from the initial event, rendering revascularization strategies inadequate in preventing future events [1]. And finally, clinical outcomes for SCAD patients undergoing percutaneous coronary intervention are less predictable due to the technical challenges inherent to the disease. Therefore, in patients who are clinically stable without high-risk anatomy, conservative management with close inpatient monitoring should be pursued.

## Decision to Proceed to Intervention and Challenges

While most cases of SCAD can be successfully managed conservatively, a minority of patients require more aggressive therapy. In the patient with an occluded vessel, evidence of ongoing cardiac ischemia, arrhythmia, or hemodynamic compromise coronary intervention to re-establish flow should be considered.

When addressing lesions, interventionalists should maintain a conservative approach. Instead of attempting to reinstate normal coronary structure, the priority should be to achieve TIMI flow grade 3 alone due to the increased risk for additional iatrogenic dissection or vessel occlusion associated with the treatment of SCAD [5, 26•, 33].

Because of the etiology of the vascular obstruction and possibly due to the high coincidence of arteriopathies in patients with SCAD, higher rates of technical failure and procedural complications are observed in comparison to patients undergoing PCI for atherosclerotic disease. Iatrogenic coronary artery dissection occurs in 3–4% of patients, compared to < 0.2% in all other PCI events, and periprocedural complication rates have been reported as high as 22% in small cohorts [15, 33]. In one third of cases, IMH propagation may occur resulting in unplanned stent placement [5, 17]. Failure of percutaneous intervention (PCI) has been reported to occur in studies ranging from 30 to 53%, depending on the criteria utilized. Patients undergoing percutaneous intervention also experience a significantly higher risk, over sixfold, for emergent coronary artery bypass graft (CABG) due to PCI failure. This was observed in all groups regardless of observed vessel patency on initial angiography [17]. While rare, stent malposition after IMH resolution has been reported [34]. Stent placement was not associated with a reduced risk for SCAD recurrence [17].

Described techniques to minimize risk for iatrogenic complications during PCI include the use of small-caliber balloon angioplasty or cutting balloon to fenestrate the intimal-medial membrane to depressurize the false lumen. Proximal and distal edge stent placement to minimize IMH propagation is recommended [15, 21]. The use of bioresorbable coronary scaffolds has been suggested to reduce complications [15, 35, 36]. When stenting is necessary, second-generation drug-eluting stents (DES) are advised due to lower rates of future major adverse cardiac events when compared to bare metal stents (BMS) [15, 26•, 37].

CABG is reserved as a bail-out method for patients experiencing PCI failure or exceedingly poor interventional options [15]. Due to the infrequent use of CABG in SCAD, data is scarce. Existing cases have shown frequent graft failure at follow-up, likely due to competing flow following native vessel healing [17]. Optimal management is not established for patients with clinically stable high-risk anatomy, such as those with left main or multivessel proximal disease. CABG should be considered in these patients, though data on outcomes for this patient subset is lacking [5, 21]. Fortunately, the overall mortality of SCAD patients remains low.

## Conclusion

SCAD is an increasingly recognized entity for ACS in young, female patients. While the pathogenesis remains elusive, sex hormones and underlying systemic vascular disorder, specifically FMD, have been implicated. Patients with SCAD should be routinely screened for EVA abnormalities due to the risk for associated intracerebral aneurysms and other vascular abnormalities which necessitate intervention or carry implications for future pregnancy. Further work is ongoing to understand the genetic implications of SCAD.

Management of SCAD is primarily conservative. Initial management typically involves the use of antiplatelet and anticoagulation due to ACS; however, this should be discontinued after diagnosis of SCAD is established. The use of guideline-directed medical therapy for patients with reduced ejection fraction is recommended. Beta-blockers may have additional benefits to reduce risk of future events. Studies are ongoing to determine optimal medical therapy after SCAD.

PCI is reserved for patients with hemodynamic compromise or ongoing ischemia despite conservative measures. This is due to the high risk for periprocedural complications associated with PCI in SCAD patients. Techniques to minimize risk include decompression of the false lumen through fenestration techniques with small-diameter cutting balloon angioplasty, proximal and distal end stenting, along with the use of DES when stenting is warranted. Due to the self-resolving nature of SCAD lesions, proceduralists should perform minimal intervention to achieve acceptable distal flow.
